# From Intermolecular Poses to Thermodynamics Using Subdivided Spheres

**DOI:** 10.1021/acs.jpcb.6c01665

**Published:** 2026-06-09

**Authors:** Isabel Vinterbladh, Jordan Bye, Robin Curtis, Harold Hatch, Sergei Grudinin, Mikael Lund

**Affiliations:** † Division of Computational Chemistry, Department of Chemistry, 712199 Lund University, 223 62 Lund, Sweden; ‡ Manchester Institute of Biotechnology, Department of Chemical Engineering, 5292 University of Manchester, Manchester M1 7DN, U.K.; § Chemical Informatics Research Group, Chemical Sciences Division, 10833 National Institute of Standards and Technology, Gaithersburg, Maryland 20899-8380, United States; ∥ Univ. Grenoble Alpes, CNRS, Grenoble INP, LJK, 38000 Grenoble, France; ○ LINXS Institute of advanced Neutron and X-ray Science (LINXS), Mesongatan 4, 224 84 Lund, Sweden

## Abstract

Computing molecular thermodynamic properties is instrumental in multiple scientific disciplines, such as statistical physics, *N*-body simulations, and molecular docking. However, exact thermodynamic calculations are almost always not feasible. In this work, we introduce a versatile algorithm designed to rapidly compute the two-body partition function, its related thermodynamic properties, and the second virial coefficient for anisotropic nanoparticles and proteins under the rigid-body approximation. Our method involves constructing a quasi-regular grid in the 5D angular space between pairs of arbitrary objects and efficiently scanning the radial-angular space between the rigid molecules. Where available, we find excellent agreement with light and X-ray scattering experiments, as well as with Monte Carlo simulations. Our results suggest a correction to current coarse-grained protein force fields, and we further discover a new, counterintuitive effect of temperature on virial coefficients, caused by a population shift in angular space due to the dielectric response of water. Finally, the grid can serve as an interpolation table for *N*-body simulations, increasing their performance by orders of magnitude.

## Graphical abstract

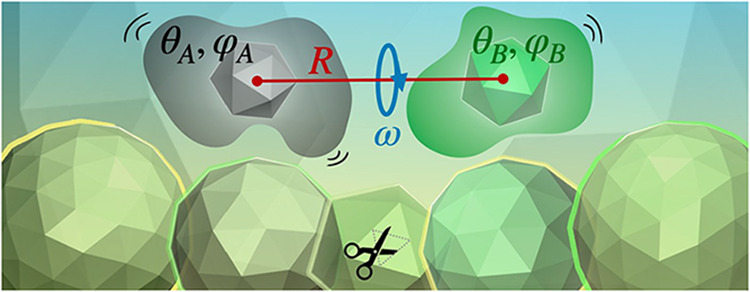

## Introduction

Intermolecular interactions in macromolecular solutions are central to biopharmaceutical development,^1–4^ where formulation stability determines product viability and shelf life;^5^ to food science, where protein functionality governs texture and processing outcomes;^6^ and to biophysics, where protein condensation underlies membraneless organelle formation and pathological aggregation.^7^ Understanding these interactions at the two-body level is essential for studying larger many-body systems, regardless of whether they are patchy particles, proteins, or other assemblies. The osmotic second virial coefficient, *B*_2_, exactly represents an ensemble average over all two-body microstates and is a commonly used measure to gauge intermolecular interactions in solution. Since the seminal work of George and Wilson,^8^ who identified a narrow *B*_2_ window favorable for protein crystallization, the second virial coefficient has been widely adopted as a predictor of solution behavior. Importantly, proteins are not isotropic spheres but rather anisotropic, patchy colloids,^9^ and both small-angle scattering^10,11^ and coarse-grained modeling^12^ show that the orientational dependence of the pair potential directly influences *B*_2_.

*B*_2_ and other thermodynamic properties can be obtained from the two-body partition function by integrating over one radial and five angular dimensions,^13^ the latter describing the relative orientation of the two interacting bodies. Early scattering experiments by Velev et al.^14^ demonstrated that electrostatic anisotropy directly affects measured *B*_2_ values for chymotrypsinogen, underscoring the need for models that explicitly resolve orientational degrees of freedom. In this work, we adopt the rigid-body approximation: globular proteins possess stable tertiary structures, and internal degrees of freedom can be considered preaveraged in coarse-grained representations. Under this assumption, the remaining challenge is exhaustive sampling of the five-dimensional angular space. Molecular-dynamics-based free energy methods—umbrella sampling, thermodynamic integration, free energy perturbation^15–17^ —can in principle compute potentials of mean force and thus *B*_2_, and Lopez et al.^4^ established rigorous connections between MD subvolume analysis and virial coefficients. However, exhaustive orientational sampling of macromolecules with such methods remains computationally prohibitive. Alternative computational strategies include Fast Fourier transforms to accelerate *B*_2_ calculations for atomistic protein models,^2^ and precomputed lookup tables in angular space combined with data-driven approaches such as neural network potentials for anisotropic nanoparticles.^18^

Efficient discretization of orientational space is a challenge since uniformly distributing points on a sphere remains an unsolved mathematical problem.^19^ Fibonacci spiral methods provide near-uniform distributions but irregular neighbor connectivity complicates interpolation. Lebedev quadratures offer optimal integration efficiency for smooth functions and are used in quantum chemistry, while HEALPix tessellation provides hierarchical equal-area partitioning.^20^ For the full rotation group SO(3), optimal incremental grids using Hopf fibration have been introduced.^21^ Blech et al.^22^ recently reviewed spherical quadrature methods for molecular physics. Recently, multivariate polynomial interpolation using Chebyshev and trigonometric basis functions to approximate anisotropic pair potentials from limited training data was demonstrated.^23^

Here we present a framework for fast and robust calculation of the two-body partition function and, subsequently, all related thermodynamic quantities.^24^ Our approach rests on two assumptions: (i) the rigid-body approximation, where internal bonds and internal interatomic distances are fixed; (ii) a finite angular sampling rate. The multidimensional angular space is discretized using subdivided icosahedrons that generate a quasi-regular grid in 5D angular space with guaranteed neighbor connectivity, suitable for both numerical integration and barycentric interpolation. This grid-based strategy differs from the polynomial interpolation of Fakhraei et al.^23^ by guaranteeing neighbor connectivity at every resolution; moreover, the grid data can serve as training input for such data-driven approaches.

We apply our model to calculate *B*_2_ for simplified charged patchy particles and coarse-grained protein models; nevertheless, the framework is general and readily extendable to study a wide variety of two-body systems. The framework achieves excellent agreement with both Monte Carlo sampling and experimental light scattering measurements. Our results reveal a previously undocumented counterintuitive temperature effect on *B*_2_ arising from the dielectric response of water, and suggest corrections to current protein force fields for capturing electrostatic anisotropy in patchy charged systems. Implemented in the open-source package Duello,^25^ the model provides a general method for connecting coarse-grained molecular models to the physics of protein solutions, highlighting the interplay between anisotropic forces and thermodynamic observables.

## Methods

### Rigid Two-Body Configurational Integral

Figure 1A shows the six-dimensional (6D) space required to describe a pose between two rigid bodies: The space consists of the mass-center distances, *R*, and a set of angles **Ω** = ω, θ_*A*_, ϕ_*A*_, θ_*B*_, ϕ_*B*_. The ω is a torsion angle, while θ and ϕ represents two sets of spherical angles for A and B respectively, see Supporting Information (SI). Knowing the potential energy, *V*(*R*, **Ω**), we can in principle calculate any thermodynamic function by fully exploring intermolecular poses using the partial two-body configurational integral

1 $$Z\left(R\right)=\int e^{-\beta V\left(R,\Omega \right)}d\Omega /\int d\Omega$$

where β = 1/(*k*_B_ *T*) is the inverse thermal energy. This formidable integral has no general analytical solution, and we need to resort to numerical techniques. The main novelty of this work is the design of an almost regular grid in Ω-space, which allows (1) fast and robust evaluation of thermodynamics, and (2) tabulation and interpolation of 6D pair-potentials. The latter greatly accelerates many-body simulations^23,26^ of macromolecules, including proteins.^3,27^

**Figure 1:**
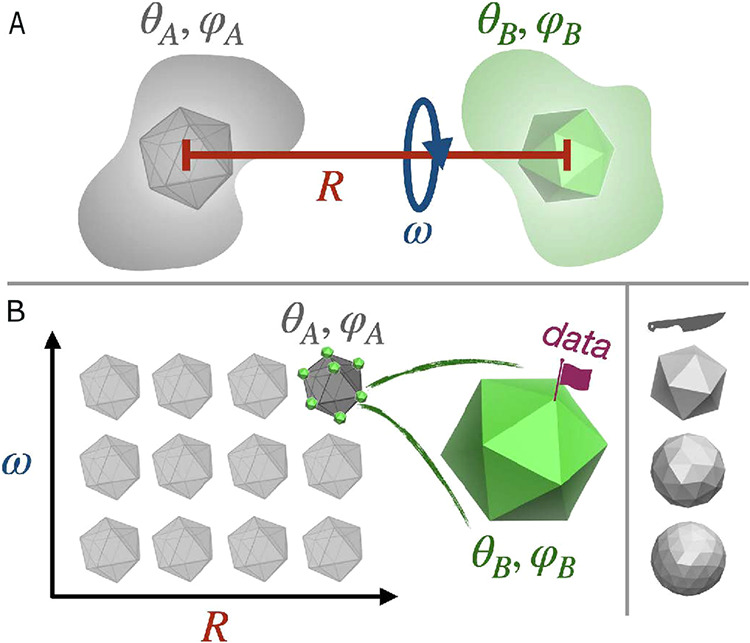
(A) Representation of the 6D space (*R*, ω, θ_*A*_, ϕ_*A*_, θ_*B*_, ϕ_*B*_) capturing all poses of two rotating, aspherical molecules, A and B. (B) Nested 6D table structure where center–center separation, *R*, and dihedral angle, ω, are stored on a uniform 2D grid. Remaining orientations of A are represented by (gray) icospheres where each vertex holds another (green) icosphere representing the orientation of B. Associated data is stored on the vertices of the latter (green) icospheres. The right-most section illustrates repeated spherical subdivision of the icosahedron^28,29^ to increase angular resolution.

The challenge lies in evenly distributing the two sets of azimuthal and polar angles (θ_*A*_, ϕ_*A*_, θ_*B*_, ϕ_*B*_) on two unit-spheres. Analytically distributing an arbitrary number of points uniformly on a sphere is an unsolved problem in mathematics.^30–32^ The Golden Spiral or Fibonacci Sphere method are good approximations,^19,33^ but the number of neighbors is irregular, which complicates interpolation of the points’ values.

Convex regular polyhedrons—also known as Platonic solids—have congruent faces and the vertices are evenly distributed on a sphere,^28,29^ making them perfect placeholders for (θ, ϕ). The *regular icosahedron*, which has *n*_*f*_ = 20 faces and *n*_*v*_ = 12 vertices, can be subdivided^28,29^ to construct additional points on a sphere, by which we obtain *n*_*v*_ = 2 + 10·(*n*_*d*_ + 1)^2^, for *n*_*d*_ subdivisions (see Figure 1, bottom right). Although, nearest neighbor distances of subdivided vertices fluctuate slightly (Table S1) depending on the subdivision method, each vertex is always connected to five or six neighbors, which is advantageous for tabulation and interpolation.

The discretized partial configurational integral is,

2 $$Z\left(R\right)\approx \frac{1}{\sum g_{\Omega }}\sum_{\omega }\sum_{\theta_{A}\;}\sum_{\phi_{A}\;}\sum_{\theta_{B}\;}\sum_{\phi_{B}\;}g_{\Omega }\times \exp\left\{-\beta V\left(R,\underset{\Omega }{\underset{︸}{\omega ,\theta_{A}\;,\phi_{A}\;,\theta_{B}\;,\phi_{B}\;}}\right)\right\}$$

which converges to eq 1 for large *n*_*d*_. If we can distribute ω, θ_*A*_, ϕ_*A*_, θ_*B*_, ϕ_*B*_ regularly on two unit spheres, using *N* grid points in (θ_*A*_, ϕ_*A*_) and (θ_*B*_, ϕ_*B*_); and *M* points in ω, the configurational subvolume *g*_Ω_ = (4π/*N*)^2^·(2π/*M*) is the product of two spherical areas with a torsion angle. For the undivided icosahedron, *g*_Ω_ is thus a constant, since all vertices are perfectly regular in (θ, ϕ). However, subdivision leads to slight fluctuations in face areas (Table S1) which is accounted for by giving each vertex a degeneracy proportional to the average spherical area of the adjoining faces, whereby *g*_Ω_ ∝ *a* (θ_*A*_, ϕ_*A*_)· *a* (θ_*B*_, ϕ_*B*_)·(2π/*M*).

While all thermodynamic functions can be calculated from Z(R) , we here limit the discussion to the potential of mean force (PMF),

3 βw(R)=−ln⁡Z(R)

and *B*_2_.^24^ The latter is available from light scattering experiments and provides a direct link between theory and measurements. *B*_2_ reports on exactly two-body interactions:^1,2,4,11,13,24,34^

4 B2=−12∫Ω∫0∞(e−βV(R,Ω)−1)4πR2dRdΩ/∫ΩdΩ=−2π∫0∞(Z(R)−1)R2dR=B2hs−2π∫σ∞(Z(R)−1)R2dR

where ∫_**Ω**_d**Ω** = 32π^3^ since ∫_0_^2π^dω = 2π and ∫_0_^π^∫_0_^2π^sinθ dϕdθ = 4π. *B*_2_^hs^ = 2πσ^3^/3 is the hard-sphere contribution where σ is a radial distance of closest approach where *w*(*R* < σ) = ∞ is assumed. Note that for aspherically shaped soft bodies, the choice of σ, and thus B_2_^hs^, is operational. For systems with net attractive interactions, the dissociation constant, *K*_*d*_, for the equilibrium process AB ⇌ A + B can be estimated by^35^ *K*_*d*_^–1^ = −2*N*_*A*_(*B*_2_ – *B*_2_^hs^).

### Two-Body Pose Exploration

The following details one of many ways to explore all rigid body poses using subdivided spheres. Consider two icospheres, A and B, both centered on the *z*-axis and with a center–center separation, *R*. Each icosphere consists of a set of unit vectors representing *n*_*v*_ vertices that form a (quasi-)­regular grid in (ϕ, θ). We construct three rotational transformation operations:

1. Rotate A so that vertex *i* aligns with the *z*-axis.
2. Rotate B so that vertex *j* aligns with the negative *z*-axis. By now, a pair of *i*–*j* vertices point toward each other.
3. Rotate B around the *i*–*j* connection line (*z*-axis) by Δω.

Transformation (1) is applied to a reference structure of molecule A and operations (2) and (3) to a reference structure of molecule B. For all *n*_*v*_^2^ aligned vertex pairs, (3) is repeatedly applied to B in order to explore torsion angles, ω. This procedure, trivially parallelizable, is carried out for all *R*. (1) explores ϕ_*A*_, θ_*A*_; (2) explores ϕ_*B*_, θ_*B*_, while (3) explores ω, see Figure 1. Finally, we note that the 6D grid can be explored using Metropolis Monte Carlo importance sampling.^27^ Compared to continuous space, this has the advantage that configurational space is known, whereby revisiting a previous state is computationally cheap due to energy caching. By assuming that nonsampled states have zero weight, grid based MC sampling would still provide Z and full access to thermodynamics.

### Coarse Grained Protein Model

To calculate *B*_2_ for globular proteins using structural information, we use the Calvados3 model^36,37^ where each amino acid is coarse grained to a single interaction site. The site–site pair energy consists of a short-range Ashbaugh-Hatch (AH) potential^36,38^ and a screened Coulomb (SC) potential to capture exchange repulsion; short-range attraction; and electrostatics in an implicit solvent and salt medium. Assuming pairwise additive interactions between residues *i* and *j* on the two proteins, A and B, the total pose energy is

5 $$V\left(R,\Omega \right)=\sum_{i}^{N_{A}}\sum_{j}^{N_{B}}u_{\mathrm{ij}}^{\mathrm{AH}}+u_{\mathrm{ij}}^{\mathrm{SC}}$$

We propose two modifications to the Calvados3 model to improve qualitative agreement with measured osmotic second virial coefficients for globular proteins:

1. Off-center side-chain charges
2. Centered, but polarizable side-chain charges

In variant (1), charged atoms are maintained at the original all-atom representation positions, instead of moving them to the mass-center of the residue. The additional charge site has the Ashbaugh-Hatch parameters σ = 2.0 Å and λ = 0. The interaction strength, ϵ is the same as for all other sites. In variant (2), the original Calvados3 model is maintained, but we assign an operational, isotropic polarizability, α to each amino acid with a charged side-chain. We then add to eq 5 the following ion-induced dipole^39^ pair-potential to the site–site energy:

6 $$u_{\mathrm{ij}}^{\mathrm{pol}}=-\frac{1}{2}\left(\left|E_{i}{|}^{2}\alpha_{j}+\right|E_{j}{|}^{2}\alpha_{i}\right)=-\frac{\left(q_{i}^{2}\alpha_{j}+q_{j}^{2}\alpha_{i}\right)}{8\pi \epsilon_{0}\epsilon_{r}}{\left(\frac{1}{r_{\mathrm{ij}}^{2}}+\frac{\kappa }{r_{\mathrm{ij}}}\right)}^{2}e^{-2\kappa r_{\mathrm{ij}}}$$

where we have used the field originating from a screened Coulomb potential with inverse Debye screening length, κ. Pair-wise additivity is crudely assumed, which is commonly done also for other (induced) dipole interactions, i.e., 1/*r* ^6^ and, like Lennard-Jones parameters, α is of empirical nature.

### Metropolis Monte Carlo Simulations of Charged Patchy Particles

To test the subdivision approach, we also use a spherical, charged patchy particle (CPP) model^40^ to mimic charged, colloidal particles. Here the AH potential in eq 5 use λ = 0 whereby it equals the purely repulsive Weeks-Chandler-Anderson (WCA) potential.^41^ To verify that the subdivision approach, eq 2, gives correct thermodynamics, we compare with PMFs obtained from continuous space Metropolis-Hastings Monte Carlo simulations^3,42^ of two CPPs. The PMF, *w*(*r*), is sampled using histogram binning.

### Static Light Scattering Experiments

We carried out static light scattering experiments to determine second virial coefficients (*B*_2_) for lysozyme and Cgn in 10 mM TRIS buffer at pH 7, with varying concentrations of sodium chloride. Protein solutions were prepared and analyzed following the procedure described by Bye et al.,^43,44^ using a Zimm plot analysis to extract *B*_2_ values.

## Results and Discussion

### Free Energy of Patchy Particles

Now in position to explore the partition sum, Z(R) , we proceed to investigate the interaction free energy or PMF, eq 3 between two simplified charged, patchy particles (CPP) with properties mimicking globular proteins.^40^ The spherical CPP, *P*_8_^1^ particle,^40^ has a net charge of −8*e* and a single positively charged patch (see inset of Figure 2, top), effectively giving it a high dipole moment. The PMF shown in Figure 2 reveals both short-range attraction and long-range repulsion due to the combination of an electric monopole and dipole. For reference, we have also calculated the PMF using Metropolis-Hastings^42^ Monte Carlo (MC) simulations with β*w*(*R*) = −ln *g*(*R*) where *g*(*R*) is the sampled radial distribution function for a pair of *P*_8_^1^ particles.

**Figure 2:**
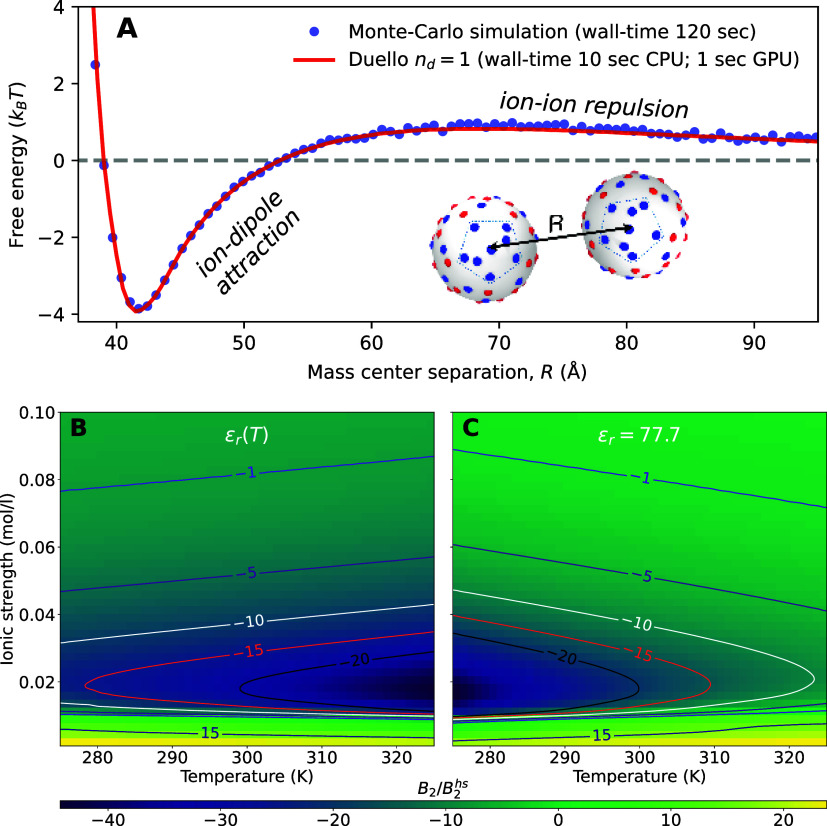
(A) Angularly averaged interaction free energy, *w*(*R*), along the center–center separation, *R*, for two *P*_8_^1^ patchy particles. Calculated using Metropolis-Hastings MC sampling (symbols) with roto-translational trial moves, and from the partition function, eq 2, with an angular resolution of 0.37 rad (line). Pairwise interactions between individual beads is a combination of soft repulsion (WCA)^41^ and a screened coulomb potential with a Debye screening length of 43 Å (5 mM ionic strength). The inset shows the two nanometer-sized *P*_8_^1^ patchy particles.^40^ (B–C) Reduced *B*_2_^*^ = *B*_2_/*B*_2_^hs^ for *P*_8_^1^ over varying ionic strengths and temperatures. The left-hand side includes a temperature dependent dielectric constant, while on the right-hand side, ε_*r*_ is artificially fixed to that of water at 300 K. Contour lines are constructed by interpolation between 2408 *B*_2_ values.

With a sufficient number of subdivisions, *n*_*d*_, the discrete 6D model should approach that of a continuous angular space, eq 1. While continuous-space Metropolis Monte Carlo should therefore yield identical thermodynamics, we note the following technical differences: (1) MC preferentially visits low-energy states and may require enhanced sampling techniques for highly attractive interactions;^45,46^ (2) when calculating the virial coefficient (or other volume integrals), the *R* ^2^-weighting amplifies sample noise at large separations and convergence requires long MC simulations, particularly if the tail is poorly sampled due to the previous point; (3) for coarse grids, the 6D table approach may miss highly specific and attractive intermolecular orientations.^3^ Although this is also a risk for continuous-space MC, the Metropolis-Hastings algorithm steers toward such poses, provided that they are not surrounded by barriers significantly larger than the thermal energy. For the tabulation approach, one remedy is to use a finer angular grid at short interparticle distances. Alternatively, if the low energy pose is known, e.g., from crystallography, the angular grid may be designed so that the orientation is guaranteed to be precomputed; (4) For small systems, MC has poor parallel scaling since each state depends on the previous. In contrast, the grid based integration can be evenly distributed among all available CPU or GPU cores (see wall-times in Figure 2A), where we used a 10 CPU core ARM architecture with integrated GPU acceleration. Note that we have used the same number of configurations in MC and on the grid, and the speedup is mainly from improved parallelism.

Figure 2B–C, shows how *B*_2_ for *P*_8_^1^ particles varies with ionic strength and temperature. For plot 2B, the dielectric constant is a function of temperature, ϵ_*r*_(*T*), which affects both the Bjerrum and Debye lengths. Plot 2C shows the same calculations, but with a fixed dielectric constant, ϵ_*r*_(*T* = 300 *K*) = 77.7. Thus, in this artificial case, temperature affects only the thermal energy but not the dielectric properties of the medium. As previously observed,^44,47^ an uneven surface charge distribution may lead to nonmonotonic dependence of interparticle interactions with ionic strength, *I*. At very low *I*, the monopole-monopole repulsion, which is the leading term in a multipole expansion, dominates whereby the virial coefficient is large and positive (yellow region in Figure 2B–C). In contrast, at high salt concentrations, electrostatic interactions are mostly screened (green region). However, in between, at intermediate ionic strengths, *attraction* (*B*_2_ < 0) is observed due to favorable multipolar alignment, e.g., due to ion-dipole interactions (dark blue region). Interestingly, the attractive patch interaction at intermediate ionic strengths, is significantly enhanced at elevated temperatures. To the best of our knowledge, this *T*-variation is undocumented and may at first seem counterintuitive, as higher temperatures should flatten the probability distribution in angular space, thereby weakening orientationally dependent interactions. However, this is counteracted by the water dielectric constant that *decreases* with temperature, thus increasing electrostatic energies. In other words, for water, the Bjerrum length, λ*_B_* (*T*) ∝ (ε_*r*_(*T*) *k*_B_ *T*)^−1^ is an increasing function of *T*. The Boltzmann factor will, in turn, favor poses with attractive interactions, leading to more alignment at elevated temperatures. As a final intricacy of effective potentials, the Debye length λ*_D_* (*T*) ∝ λ*_B_* (*T*)^−1/2^ decreases slightly with temperature, meaning that salt screens more efficiently.

### Globular Proteins

We now investigate the *B*_2_ for two non-spherical, globular proteins, taking into account secondary structural information from the rigid protein. The protein–protein interaction energy, *V*(*R*, **Ω**), is estimated using the Calvados3 model^36,37^ where each amino acid is treated as a single interaction site, see Methods section. The model accounts for site-specific short-range attraction and electrostatics are treated using linearized Debye–Hückel theory, see eq 5. Figure 3, top shows how *B*_2_ for lysozyme (*M*_*w*_ = 14.3 kDa, PDB ID 4LZT) varies with ionic strength. Lysozyme, being highly positively charged at pH 7, has a high positive *B*_2_ at low ionic strength, reflecting electrostatic repulsion. Adding salt, this is significantly screened and *B*_2_ drops. The experimental trend is well captured by the Calvados3 model.

**Figure 3:**
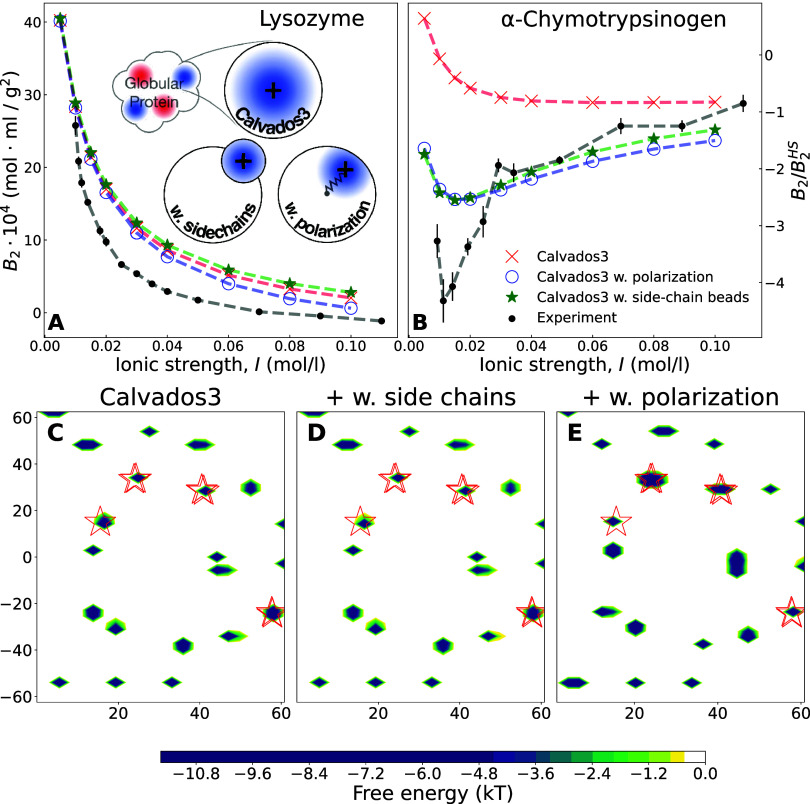
Top: Calculated and measured^44^ *B*_2_ versus ionic strength, *I*, at neutral pH for lysozyme (A) and Cgn (B). Three variations of the Calvados3 interaction model^36,37^ used, see illustration on left plot. Icosahedrons with *n*_*d*_ = 4 subdivisions were used to construct angular space. Bottom: PCA results for respective Calvados3 model for Cgn plotted. (C) with vanilla Calvados3, (D) with added side-chains and (E) with added polarization. Red stars identify poses of experimental crystal structures.

At pH 7, α-Chymotrypsinogen A, “Cgn” (*M*_*w*_ = 25.3 kDa, PDB ID 7KTZ), has a net-charge of +4*e* and a highly uneven, “patchy” charge distribution.^34,47^ The latter results in an attractive electrostatic interaction that have a maximum at intermediate (low) ionic strengths.^34,44,47,48^ Adding more salt, screens the attraction and *B*_2_ increases. The vanilla Calvados3 model^36,37^ approximates well the virial coefficient at high ionic strength, but fails to capture the nonmonotonic variation at low ionic strength. The qualitative discrepancy is because the model centers charges in the middle of amino acid residues, leading to an underestimated electrostatic attraction. A simple remedy is to instead place charged side-chains at their original position from the all-atom original structure, see inset of Figure 3A. This recovers the nonmonotonic trend observed experimentally.

Calvados3 was developed to model intrinsically disordered proteins where explicit sampling over side-chain positions is impractical.^36^ To maintain a central charge while capturing the effect of being off-center, one option is to attach the charge in an implicit spring to mimic configurational polarization due to surrounding charges that either attract or repel the side-chain. This is equivalent to an always attractive ion-induced dipole interaction energy, $u=-\frac{1}{2}|E{|}^{2}\alpha$ where **E** is an external field and α is a polarizability reflecting side-chain flexibility. If the field originates from an unscreened point charge, the energy contribution decays as 1/*r* ^4^, see eq 6. Note that we assume pairwise additivity for the ion-induced dipole terms. The same is commonly done for van der Waals interactions (1/*r* ^6^) and, much like Lennard-Jones parameters, α is best regarded as a heuristic parameter used to reintroduce additional electrostatic attraction.

As can be seen in Figure 3B, this polarization model has a similar effect as using explicit side-chain charges for Cgn at low ionic strength. For the mainly repulsive lysozyme, where close-contact configurations are sparsely populated, the two alternative interaction models have minute effects, particularly at low ionic strength.

The configurational space of Cgn is used in a Principal Component Analysis (PCA), where clusters of similar poses at free energy minima are found. The darker the cluster, the higher density of configurations present and a lower free energy is observed. The red stars represent poses corresponding to experimental structures,^49^ which nicely fit the PCA clusters. This shows that Duello indeed measures empirical minimum configurations. Additionally, as the polarization model displays the largest clusters, it confirms that the model captures more interactions than the vanilla version. For further discussion, see SI.

### 6D Interpolation

Many-body simulations of globular proteins^26,50^ benefit from energy lookup tables in angular space.^3,27,51^ In numerical calculations, such as molecular dynamics and MC simulations, pair-wise interactions is a bottleneck, and efficient lookup tables will accelerate simulation times. Precomputing interaction energies, *V* (or any other property) for fixed values of the center separation radius, *R*, and the dihedral angle, ω (Figure 1), the remaining angles (θ_*A*_, ϕ_*A*_, θ_*B*_, ϕ_*B*_) are converted to normalized barycentric coordinates^52^ on body A (λ_1_^*A*^, λ_2_^*A*^, λ_3_^*A*^) and on body B (λ_1_^*B*^, λ_2_^*B*^, λ_3_^*B*^). The proposed interpolation is:

7 $$V\left(R,\Omega \right)={\left(\lambda^{A}\right)}^{T}V^{\mathrm{AB}}\lambda^{B}={\left[\begin{array}{l} \lambda_{1}^{A}\\ \lambda_{2}^{A}\\ \lambda_{3}^{A} \end{array}\right]}^{T}\left(\theta_{A},\phi_{A}\right)\left[\begin{array}{lll} V_{11} & V_{21} & V_{31}\\ V_{12} & V_{22} & V_{32}\\ V_{13} & V_{23} & V_{33} \end{array}\right]\left(R,\omega \right)\left[\begin{array}{l} \lambda_{1}^{B}\\ \lambda_{2}^{B}\\ \lambda_{3}^{B} \end{array}\right]\left(\theta_{B},\phi_{B}\right)$$

where ***V*** ^*AB*^ is generated from tabulated data on the two faces (3 × 3) containing **λ** ^*A*^ and **λ** ^*B*^. Each interpolation in this 4D subspace requires 3 × 3 vertex look-ups. If the remaining *R* and ω dimensions are stored on a 2D array, see Figure 1, bottom, the interpolation involves a total of 2^2^·9 = 36 data points. This is significantly less than the 2^6^ = 64 points required if all six dimensions are stored on a 6D array. While the SI explains more, further investigation of 6D interpolation is left to future studies.

## Conclusion

We have developed a computationally efficient method that explicitly considers orientationally dependent intermolecular energies, which is required for predicting solution thermodynamics of anisotropic molecules. We have shown that the pair interaction between arbitrarily shaped molecules can be represented by a 6D table based on vertices of subdivided icosahedrons, representing a tessellated unit sphere where the triangular faces provide convenient interpolation using barycentric coordinates, frequently used in, e.g., computer graphics.

Increasing the number of subdivisions, results converge toward the exact numerical solution obtained from Metropolis-Hasting MC sampling. In addition, to a much improved angular regularity, 6D barycentric interpolation using nested icospheres, maintaining uniform sampling on the unit sphere, leads to a significantly reduced number of table lookups (*n* = 36) compared to interpolation in a regular grid (*n* = 64). Moreover, the algorithm is well suited for parallel execution on both CPUs and GPU and is typically 1-2 orders of magnitude faster than Metropolis Monte Carlo sampling.

The accuracy of using a discrete grid to evaluate the two-body partition sum and related thermodynamic functions is tested, using macromolecules with nonuniform charge distributions. Variations of Calvados3 ^36,37^ were applied on the protein Cgn, showing that to capture the nonmonotonic variations at low salt, the amino acid charges have to be placed off-center either explicitly or implicitly. Because of its automatic nature, the algorithm (Duello^25^) is suited for optimizing future protein force fields against experimental thermodynamics such as virial coefficients. Applied to protein–protein interactions, we observe a surprising attraction at elevated temperatures; the decreasing dielectric constant of water strengthens electrostatic alignment more efficiently than the thermal energy disrupts it. This interplay between molecular interactions and solvent response shows why exhaustive orientational sampling is important to connect force fields to solution thermodynamics.

The framework enables two immediate avenues. First, tuning and developing force fields via thermodynamic properties, and second, to use the presented 6D table of precomputed pair-energies in *N*-body simulations, which requires tracking pairwise orientations and efficient table lookups, developments that could accelerate coarse-grained protein simulations significantly. More broadly, the icosphere-based discretization of angular space provides a general numerical tool applicable where orientational averaging governs macroscopic behavior.

## Supplementary Material



## Data Availability

Input files and electronic notebooks are provided at Zenodo, https://doi.org/10.5281/zenodo.20485286. Open-source software for scanning 6D space and calculating PMF, B2, and Kd between rigid molecules is available at 10.5281/zenodo.15772003.^25^ An online WebAssembly version of Duello is available at https://github.com/mlund/duello.

## Notes

Absolute error or the electric potential outside a charged, patchy particle (CPP) and at different ionic strengths; Effect of angular resolution on the CPPM potential of mean force; projection of the Boltzmann weighted poses on the two PCA components are plotted for the respective Calvados version; projection of the Boltzmann weighted poses on the two PCA components for Calvados3 with polarization are displayed; showing how a 180° rotation of half of the projection on the PCA data matches the degenerate poses to the same cluster (PDF)
