# Supplementary material for: From Intermolecular Poses to Thermodynamics Using Subdivided Spheres
Source: J Phys Chem B. 2026 Jun 9;130(27):6751–8. doi: 10.1021/acs.jpcb.6c01665 (PMC13359104; doi:10.1021/acs.jpcb.6c01665)
Supplement: Supplementary file 1 [file jp6c01665_si_001.pdf]

Supporting Information for: From Intermolecular Poses to Thermodynamics using Subdivided Spheres

Isabel Vinterbladh,[†] Jordan Bye,[‡] Robin Curtis,[‡] Harold Hatch,[¶] Sergei Grudinin,[*,§] and Mikael Lund[*,‖,⊥]

†*Division of Computational Chemistry, Department of Chemistry, Lund University, 223 62 Lund, Sweden*

‡*Manchester Institute of Biotechnology, Department of Chemical Engineering, University of Manchester, Manchester M1 7DN, United Kingdom*

¶*Chemical Informatics Research Group, Chemical Sciences Division, National Institute of Standards and Technology, Gaithersburg, Maryland 20899-8380, USA*

§*Univ. Grenoble Alpes, CNRS, Grenoble INP, LJK, 38000 Grenoble, France*

‖*Division of Computational Chemistry, Department of Chemistry, Lund University, Sweden*

⊥*LINXS Institute for Advanced Neutron and X-ray Science, Lund University, 223 62 Lund, Sweden*

E-mail: sergei.grudinin@univ-grenoble-alpes.fr; mikael.lund@compchem.lu.se

Interpolation on the unit sphere

Consider a function $f(\theta, \phi)$ defined on a unit sphere, where θ and ϕ are the polar and azimuthal angles of a spherical coordinate system, respectively. We might devise an interpo-

lation scheme where f is precalculated on an equidistant grid in θ-ϕ space, i.e., a 2D-array:

$$\begin{bmatrix} f(0,0) & \dots & f(\pi,0) \\ \vdots & \ddots & \vdots \\ f(0,2\pi) & \dots & f(\pi,2\pi) \end{bmatrix}. \tag{1}$$

The value at an arbitrary point $f(\theta,\phi)$ may then be estimated by *e.g.* linear interpolation of $2^d = 4$ points in this table. A significant issue with this approach is that the precomputed values are unevenly distributed on the unit sphere. For example, all elements in the first and last columns represent only two single values on the unit sphere, namely the poles. Figure S1A illustrates how a linear distribution of θ-ϕ maps onto the unit-sphere.

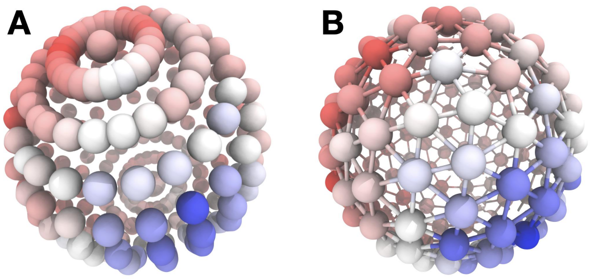

Figure S1: Points on a unit-sphere generated (A) by a uniform distribution on $\theta - \phi$ and (B) the vertices of a subdivided icosahedron, i.e. an *icosphere*. The color illustrates the value of associated data, here is an electric potential around a "P_8^1" molecule, see text.

Analytically distributing an arbitrary number of points uniformly on a sphere is an unsolved problem in mathematics.[1–3] Convex regular polyhedrons have congruent faces, and all vertices are evenly distributed on a sphere.[4,5] The *icosahedron*, which has $n_f = 20$ faces and $n_v = 12$ vertices, can be subdivided to construct additional points on a sphere, whereby we obtain $n_v = 12, 42, 92, ...$ Although the areas of the newly generated faces may differ slightly (see section below) depending on the subdivision method, each vertex is connected to exactly five or six neighbors. Interpolation at a random point $f(\theta,\phi)$ can be done by first

locating the nearest face, which is an $O(\ln n_f)$ operation if using tree partitioning.[6] Next we convert (θ, ϕ) to normalized barycentic coordinates, $\boldsymbol{\lambda} = (\lambda_1, \lambda_2, \lambda_3)$, which represent a relative position on the face. From precomputed values on the three vertices, $\mathbf{f} = (f_1, f_2, f_3)$, the interpolated value is $f(\theta, \phi) = \mathbf{1}^T(\boldsymbol{\lambda} \circ \boldsymbol{f})$, where $\circ$ is the element-wise (Hadamard) product. Barycentric interpolation requires merely three reference points, compared to $2^d = 4$ for a 2D array in spherical coordinates.

Rotating Dipole in an Electric Field

As an elementary example, for which an analytical solution exists, we study a rotating dipole in an external field. This mimics, *e.g.*, unevenly charged proteins near charged objects where alignment is influenced by the dipole and higher order moments.[7] The interaction energy of a point dipole, $\boldsymbol{\mu}$ with a uniform electric field, $\boldsymbol{E}$ is given by $u = -\boldsymbol{\mu} \cdot \boldsymbol{E}$. For a rotating dipole, the free energy of interaction is obtained by a thermal average over dipolar orientations,[8] Ω:

$$\beta A = -\ln\langle e^{-\beta u(\Omega)}\rangle_\Omega = -\ln\left(\frac{\int e^{-\beta u(\Omega)} d\Omega}{\int d\Omega}\right), \tag{2}$$

where $\beta = 1/k_BT$ is the inverse thermal energy and $d\Omega = \sin\theta d\theta d\phi$. For $u = E\mu\cos\theta$, Eq. S2 can be solved exactly to give $\beta A = -\ln\{\sinh(E\mu)/E\mu\}$. If the field originates from a point charge, q, the scalar field is $E(r) = -q/4\pi\epsilon_0 r^2$, where r is the charge-dipole separation distance and ϵ_0 is the vacuum permittivity. In agreement with a two-center multipole expansion, the angularly averaged ion-dipole interaction free energy decays approximately as $1/r^4$, if weak interactions are assumed,[8,9]

$$\beta A(r) = -\ln\left(\frac{\sinh(\beta E(r)\mu)}{\beta E(r)\mu}\right) \approx -\frac{1}{6}\left(\frac{\beta q\mu}{4\pi\epsilon_0 r^2}\right)^2. \tag{3}$$

Let us now precalculate the electric energy, $u_i = E(r_i)\cos\theta$, and store it at each ith vertex of a subdivided icosahedron or “icosphere”. The interaction free energy can be calculated

by explicitly summing the partition function over the n_v vertices,

$$\beta A_{vertex}(r) = -\ln\left(\sum_i^N e^{-\beta u_i}/n_v\right). \tag{4}$$

Figure S2 shows how Eq. 4 compares against the exact solution and that low relative errors of $\sim 10^{-4}$ are obtained even with coarse angular resolutions (see inset). As expected, lower errors are obtained with more vertices, *i.e.*, with a lower angular spacing.

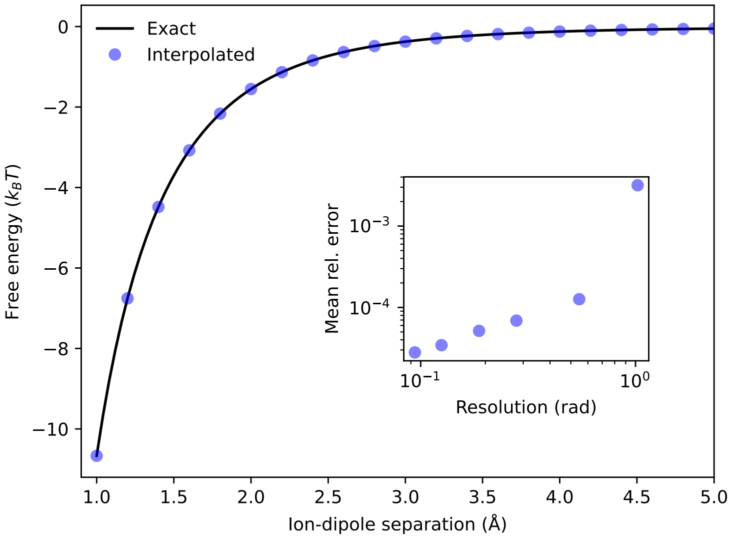

Figure S2: Ion-dipole interaction free energy obtained by a thermal average over dipolar orientations. Calculated with the exact expression, Eq. S3 (solid line); by interpolation over randomly generated points on the unit sphere ($n = 1000$ per sample). The inset shows the relative error with respect to the exact solution as a function of angular resolution, *i.e.*, decreasing number of vertices. This is calculated by summing interpolated energies from 20 randomly rotated icospheres. The relative permittivity is set to $\epsilon_r = 80$ to mimic an aqueous medium.

Electric Potential around a Patchy Colloid

In the previous example, we illustrated how icospheres can be used to tabulate the interaction of a point dipole in an external field. Since the exact solution is both known and cheaply calculated, this was mostly an exercise to illustrate the method and to gauge the

precision. We now proceed with a slightly more realistic scenario where we tabulate the electric potential around a simplified charged, patchy particle (CPP) with properties mimicking globular proteins.[10] The CPP has a net charge of $-8e$ and a single positively charged patch, see Fig. SS3, left, effectively giving it a high dipole moment. Figure S3, right, shows the electric potential 1 Å away from the surface and also illustrates how the icosphere vertices map to spherical coordinates.

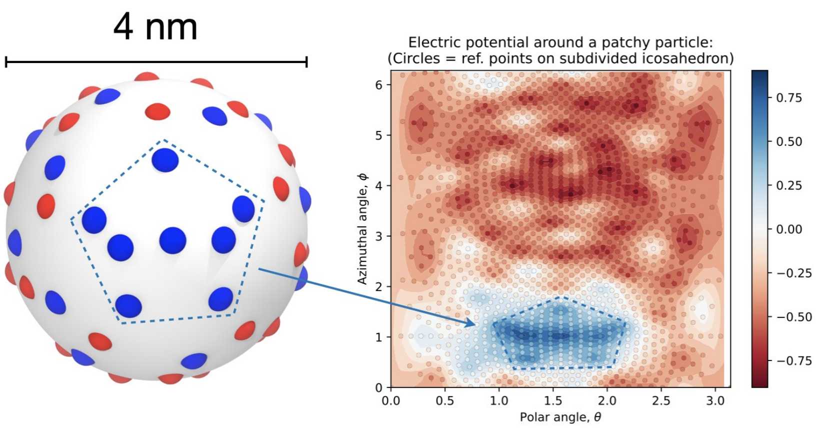

Figure S3: Left: Charged Patchy Particle Model (CPPM)[10] mimicking a globular protein. The radius is 4 nm and the surface is decorated with fixed positive and negative charges to imitate ionized amino acids. The net charge is $-8e$ and the collection of blue particles (framed by a pentagon) creates a net dipole moment of $\mu = 187\ e$Å. In the nomenclature of ref.[10] this is a "P_8^1" particle, where the superscript indicates the number of patches; the subscript the absolute net-charge. Right: Electric potential calculated at a radial distance 4.1 nm around the CPPM and mapped to spherical coordinates. Circles represent vertex positions on the icosphere and the colors in between are interpolated.

The electric potential decays with distance and salt concentration, and we now investigate how the interpolation error is affected by this. Figure S4 shows the absolute error as a function of distance and ionic strength. As expected, the error drops at large separations and high ionic strength, where the electric potential is significantly weakened.

Here we have chosen spherical coordinates parameterization with n_Ω being the total

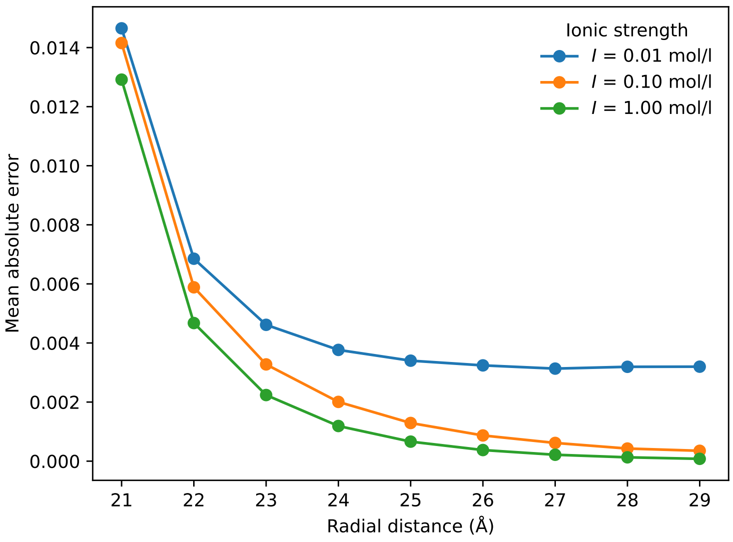

Figure S4: Absolute error or the electric potential outside a charged, patchy particle (CPP) and at different ionic strengths. Calculated using interpolation on an icosphere with an angular resolution of 0.09 radians, corresponding to 1442 vertices.

number of configurations in the spherical space, R the separation between mass centers, ω the torsion angle between the two bodies, and ϕ and θ being the azimuthal and polar angles, respectively. However, the resolution of the spherical space along R, could be adjusted such that for smaller separations the resolution is higher and for larger separations lower. This would significantly reduce the computational cost and still be able to correctly measure the potential changes, since with an increasing separation they decay rapidly and thus a coarser spherical grid would be enough. The subdivision process could even be automated by checking the gradient between faces and if it is larger than a threshold, the resolution is increased and another subdivision made. However, at the moment a uniform grid and uniform separation in R is used in Duello, and the implementation of a non-uniform spacing in the grid is part of future work.

Effect of subdivision

For the CPPM model presented in Figure 2 of the main text, we here show how the number of spherical subdivisions, n_d, affect the twobody potential of mean force. Figure S5 shows

that convergence is reached for $n_d = 1$ for this particular system, but this depends generally on the level of anisotropy of the interacting particles.

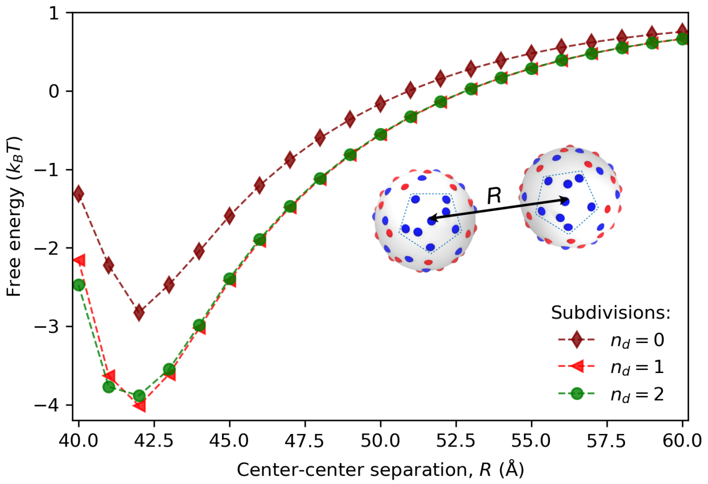

Figure S5: Effect of angular resolution on the CPPM potential of mean force. The $n_d = 0$ case corresponds to an undivided icosahedron with 12 vertices.

Face Area Fluctuations

The face areas generated by subdividing an icosahedron fluctuate slightly. As discussed in the main text, for each vertex, we calculate a weight or degeneracy, $g(\theta, \phi)$ proportional to the net spherical areas of the adjacent faces. The table below shows the standard deviation for these weights as a function of the number of subdivisions, n_d. All weights are normalized so that the average over vertices is unity.

The number of vertices for n_d subdivisions is $n_v = 2 + 10(n_d + 1)$ and the number of faces is $n_f = 20 \cdot 4^{n_d}$. The face area of a face on the icosahedron is the same as the area of an equilateral triangle face, $A = \frac{\sqrt{3}}{4}a^2$, where a is the edge length of the face.

Table S1: Degeneracy fluctuations for different subdivision levels.

n_d	0	1	2	3	4	10
std dev	0.000	0.027	0.028	0.046	0.043	0.026

Principal Component Analysis of Configurations

Free energy calculations with the developed software Duello[11] have been performed for α-Chymotrypsinogen A, "Cgn", with the original Calvados3 model as well as the two proposed modifications already discussed. In order to see if there is a connection between similar configurations and their free energy, a principal component analysis (PCA), was performed in python with scikit-learn. A trajectory with all configurations generated for the Duello calculations was used as the input to the PCA. Only frames for the minimum separation, $R =$ 41 Å, were used. The configurations are compared such that global rotation or translation of the particles would not affect the results, and thus the plots are invariant to this. Each frame represents a sample and the features are all amino acid coordinates. In other words, for one trajectory frame, which is a certain configuration of the two Cgn, the features are the center of mass xyz-coordinates of the amino acids. This gives each frame $(241 + 241) * 3 = 1446$ number of features where 241 is the number of amino acids in the Cgn structure. The number of frames being 21168 (depends on resolution chosen in Duello, for this case 0.8 was used), means that the input to the PCA is a $(21168, 1446)$ 2D-matrix of samples and features.

An unweighed 2-component dimensionality reduction PCA was executed and the results are plotted in Figure S6, upper part. Each of the three plots represent the same set of poses. A 2D-histogram of 100×100 bins of the PCA results is constructed. The calculated free energy from Duello is used as a Boltzmann weight, one Boltzmann factor for each pose where depending on the Calvados model the pose will get a different weight. The 2D-histogram is weighted with the Boltzmann factors and the PCA data is binned. Each bin represents an interval of the PCA components, the first component being the x-values and the second the y-values, such that samples with similar PCA components are binned together. The number of components in each bin is used to compute a free energy of the probability, $F = -k_B T \log(z)$, where z represents the number of occurrences in the respective bin. In this way z can be regarded as a projection of the weighted samples to the PCA space. This analysis is performed with the Boltzmann factors from respective Calvados model.

A contour plot is created with F showing the probability of certain configurations among the Duello poses. The clusters in Figure S6 represent a higher density of configurations where the free energy is at a minimum. The darker a patch is, the smaller the free energy is and the higher the probability of that configuration. The free energy has been shifted to zero, to better display comparative variations among the plots. In the plots of the upper part of Figure S6, it can be observed that there is a symmetry along the axis $x = y$ in the PCA results. This symmetry occurs because of a degeneracy in the constructed Duello poses. For each pose there is another configuration where the respective positions of the two proteins are swapped. These two poses do technically represent the same configuration, but the PCA sees them as different and therefore a cyclic 2-fold symmetry is created for the Boltzmann weighted projection on the two PCA components.

Thus, due to this degeneracy a pattern of twice as many clusters with a 2-fold symmetry though the center of the graph is constructed. If the plot is rotated by 180° degrees about the origin, the exact same pattern will be shown. Therefore, when half of the plot is rotated by 180° degrees about the origin, the clusters will coincide with the remaining data and the PCA results does not have a degeneracy splitting the data anymore. In order to perform this operation, a cut-off at $x = 0$ (or also possibly $y = 0$) divides the data and the rotation matrix,

$$R_z = \begin{bmatrix} -1 & 0 & 0 \\ 0 & -1 & 0 \\ 0 & 0 & 1 \end{bmatrix}, \tag{5}$$

is applied to the left side. Meaning, that all of the data where the x-value is smaller than zero, is rotated and added to the other half, the right side, of the plot. The lower plots in Figure S6 illustrate how the data looks like after this operation with the rotation matrix, R_z. It can be observed that the marked experimental configurations from the left side of the plot now fall upon the clusters 180° degrees from their original positions.

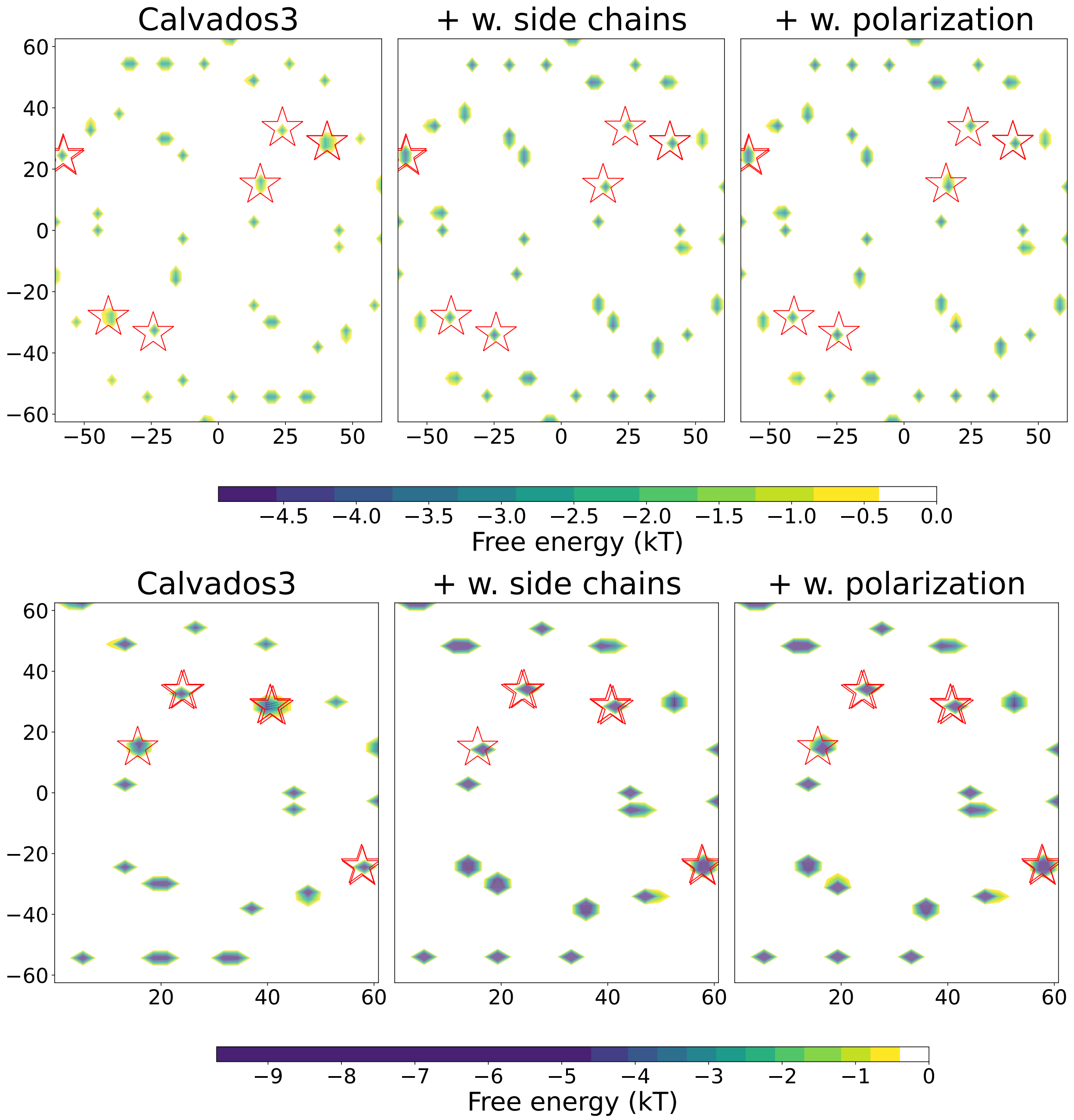

Figure S6: Top: The projection of the Boltzmann weighted poses on the two PCA components are plotted for the respective Calvados version; the original, with side chains added and with a configurational polarization parameter added. Red stars represent the poses where similar empirical structures were found. Bottom: Due to the 180° degree symmetry about the origin, the left side of the plots were rotated and added to the right side, resulting in the three lower plots. Now degenerate configurations are part of the same cluster. The red stars have also moved accordingly to represent where its structure is placed.

The first plot with the vanilla Calvados force field shows a smaller energy difference between the clusters and the less dense parts of the projection on the PCA. That the clusters have a higher free energy means that they are less dense, since a lower number of occurrences, z, gives a lower probability, F. By adding charged side chains to the model, the free energy of the clusters in the second plot is smaller, which indicates that these clusters are larger. This is also observed in the third plot, where polarization is added to the Calvados model, and thus an even higher probability of certain configurations are observed.

The red stars show where configurations represented by experimental structures have been found. Twelve different empirical structures consisting of two Cgn proteins were found through the website PPI3D[12] by using the UniProt sequence $P00766$ as input to search for related structures. These structures consist of Cgn proteins with one of the following PDB structures: $7KTY, 7KTZ, 7KU0, 7KU1, 7KU2, 7KU3$. Each pair of Cgn proteins can swap their positions, creating two experimentally valid structures for each PDB entry. These structures were compared with the Duello configurations. In order to perform a comparison, we did not take into account the separation between the two proteins, instead we were only interested in the chordal distance, which measures the angular difference. This was done by dividing the dimer structure into two single protein structures. Both structures mass-centers were shifted to origin and the same was done for all of the Duello poses. Then, each experimental protein pair was compared to respective protein from all of the Duello poses by calculating the RMSD (root-mean-square deviation), or chordal distance, between them. The smaller the value of the chordal distance is, the more similar are the experimental structure to the specific Duello pose.

For each protein pair, the Duello pose to which it had the smallest deviation was saved. Four of the experimental protein dimers corresponded to the same Duello configuration; $7KTZA - 7KTZB, 7KTYA - 7KTYB, 7KU0A - 7KU0B, 7KU3A - 7KU3B$. The first one had the smallest RMSD and was used for plotting, see Figure S7. Of the remaining complexes, $7KU0B - 7KU0A$ corresponded to a configuration having such a high energy, it

did not fit in the plotted projection on the PCA. The remaining seven structures each had a RMSD minimum, and the corresponding poses are marked in Figure S7.

For more details, see the electronic notebook `https://github.com/mlund/SI-pose-exploration`. The PCA and RMSD analysis and the exact configurations and deviations can also be found in the notebook.

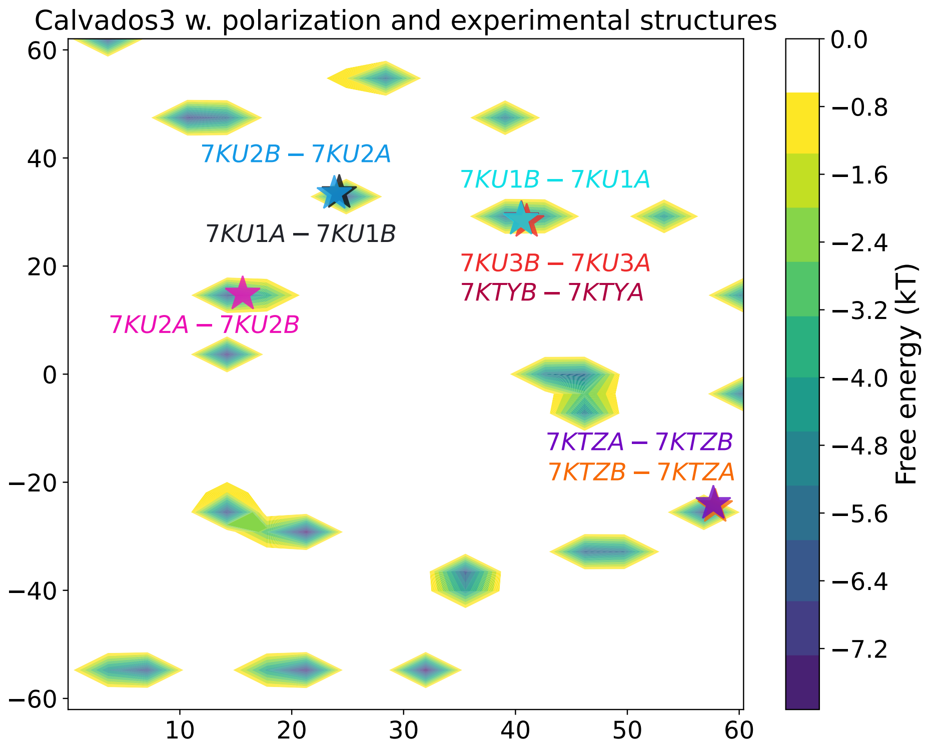

Figure S7: The projection of the Boltzmann weighted poses on the two PCA components for Calvados3 with polarization are displayed. The eight experimental structures are plotted at their best corresponding Duello configuration. All of them are on top of free energy minima. The two red structures and the light blue, are located at the same minimum, just as the orange respective purple structures. Additionally, the black and dark blue structures are also located at the same minimum. Therefore, some markers are barely visible, but all the structures visualized do correspond to different Duello poses.

Lastly, we briefly show the degeneracy among the Duello configurations to prove that the symmetry operation performed on the projection on the PCA result is valid. The configuration from frame 1 was used, and its degenerate pose was found to be frame 9317. These two poses are plotted in Figure S8 which shows how after the rotation of half of the projection on the PCA data, the poses end up at the same free minimum.

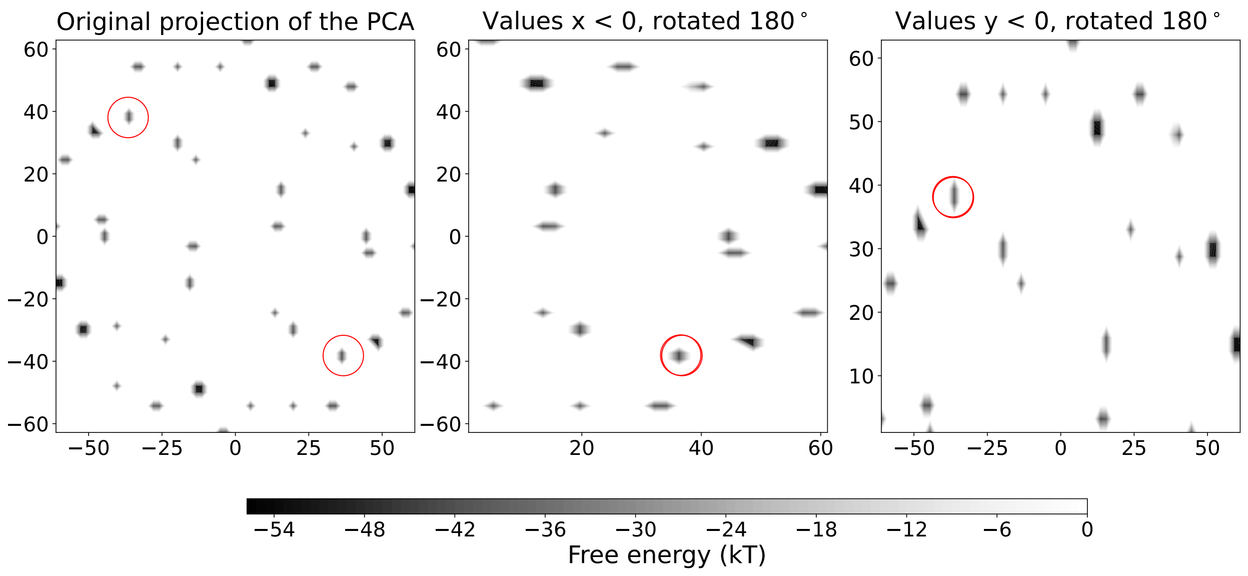

Figure S8: Showing how a 180° rotation of half of the projection on the PCA data matches the degenerate poses to the same cluster. The first plot shows the two configurations at opposite ends. Second, illustrates how the two degenerate poses now align after a 180° rotation of the values where $x < 0$. The third one, displays the same rotation, but here the rotation was applied to the values $y < 0$ and the same alignment of the degenerate poses is observed.

Rust Implementation

This is a technical brief and reflection on how the presented 6D table is implemented in the relatively new programming language *Rust*. The code and resulting command-line tool DUELLO is available at `https://github.com/mlund/duello` under a permissive open-source license.

In the main text, we define thermodynamic functions that require orientational averaging over intermolecular poses. Assuming pairwise additive potentials, each pose requires n^2 interparticle computations with $n \sim 10^3$ for, e.g., globular proteins. Depending on the angular resolution and the nature of the molecules, $10^5 - 10^6$ poses or more are required to converge the integrals. Exploring the 6D space is hence a formidable computational task, both in terms of cycles and memory usage. For this reason, we use Rust, a compiled programming language with fine-grained memory and CPU control, but with more default

memory safety and convenience functionality than, e.g., C++.[13] Examples of the latter include a default build system and dependency manager (`cargo`); unit testing framework; static code analysis and linting (`clippy`); functional programming support with advanced iterators akin to high-level languages such as Python. Icosahedron subdivision and geometric transformation functions are readily available in the Rust package system (`crates.io`) and we specifically used `hexasphere`[5] and `nalgebra`, both widely used in computer graphics software and game engines.

Due to Rusts strict data ownership rules, serial computations can often be made safely parallel with only few modifications. Specifically, the compiler forbids shared mutable memory access, thereby eliminating race conditions. When concurrently filling in the 6D table, atomic operations[14] are handled by wrapping data with `std::sync::OnceLock` from the Rust Standard Library. Sufficient for our purpose, this ensures thread-safe write access exactly once. In contrast, filling in a 6D table in classic C/C++ one would simply design a parallel nested loop that visits each element exactly once, or use opt-in atomics from C++11. The key difference is that Rust requires explicit annotation of atomic operations, and in exchange guarantees code free of race conditions. In C/C++ it is fully up to the programmer. We should mention that Rust allows unrestricted memory access by explicitly marking code blocks `unsafe`. This voids the compiler guarantees mentioned above for that section and should be used with caution. The code listing below illustrates how the 6D table is implemented in Rust using generics. C++ astute readers will be familiar with the syntax.

```
type Data           = std::sync::OnceLock<f64>; // thread-safe float
type Vec2D<T>       = Vec<Vec<T>>;              // uniform grid for R, omega
type IcoTable2D<T> = Vec<(Vertex, T)>;         // icosphere w. phi, theta (A)
type IcoTable4D<T> = IcoTable2D<(Vertex, IcoTable2D<T>)>; // phi, theta (B)
type Table6D        = Vec2D<IcoTable4D<Data>>;  // final 6D table
```

The `IcoTable2D` type associates a generic data type `T` with vertices on a subdivided icosahedron, i.e., an *icosphere*, which are readily converted to spherical coordinates, (θ, ϕ).

The table structure does leave room for improvements. For example, `Vec2D<T>` suboptimally uses separate heap allocations along the second dimension, potentially resulting in memory fragmentation. A more efficient approach would be to allocate a single contiguous memory region. This argument can be extended to the remaining table dimensions. Another more significant memory optimization, that is already used in Duello, can be made if the same icosphere is used throughout the table, i.e., if there is a uniform angular resolution. Instead of storing `Vertex` information (positions and neighbor lists) within the table, we may refer to a single, shared `Vertex` array, either via a reference counted pointer, or a static object. Despite the above short-comings, we have not detected memory-related limitations for the systems presented in this work, mainly since the tabulated data are relatively small 64-bit floating point numbers.

Possible Optimizations

The following outlines possible performance optimizations when scanning 6D space:

1. In the current implementation, pair-wise additive energies are calculated using dynamic dispatch, which in C-language translates to function pointers. While this allows for runtime flexibility through vtables, performance is affected due to lack of inlining. A static dispatch implementation would likely lead to improved performance.

2. The angular resolution, i.e., number of subdivisions, n_d is currently constant for all R. We envision an adaptive approach where we start scanning at large R with $n_d = 0$, i.e., lowest angular resolution. If the energy difference between any neighboring vertices is larger than a given threshold, n_d is incremented. The new n_d is carried over to the next R-value. Thus, by following the energy gradient in angular space, we can increase resolution only when needed, which for intermolecular interactions is at shorter separations. Due to the quadratic increase of vertices with n_d, this will bring a significant speed-up, particularly for highly charged systems at low ionic strength,

where the non-negligible energy at large separations is fairly isotropic.

3. We note that while here we integrate the 6D grid space by brute force, it is also possible to use importance sampling, i.e., Metropolis-Hastings on the proposed grid space.

References

(1) Smale, S. Mathematical problems for the next century. *The Mathematical Intelligencer* **1998**, *20*, 7–15.

(2) Cohn, H.; Kumar, A. Universally optimal distribution of points on spheres. *Journal of the American Mathematical Society* **2006**, *20*, 99–148.

(3) Leopardi, P. The applicability of equal area partitions of the unit sphere. 2024; `https://arxiv.org/abs/2408.13434`.

(4) Popko, E.; Kitrick, C. J. *Divided spheres: geodesics and the orderly subdivision of the sphere*, second edition ed.; CRC Press: Boca Raton, FL, 2022.

(5) Buhring Espinoza, P. Hexasphere: Rust sphere generation. 2024; `https://zenodo.org/doi/10.5281/zenodo.12189017`.

(6) Fuchs, H.; Kedem, Z. M.; Naylor, B. F. On visible surface generation by a priori tree structures. *ACM SIGGRAPH Computer Graphics* **1980**, *14*, 124–133.

(7) Gaspar, R.; Lund, M.; Sparr, E.; Linse, S. Anomalous Salt Dependence Reveals an Interplay of Attractive and Repulsive Electrostatic Interactions in $\alpha-$synuclein Fibril Formation. *QRB Discovery* **2020**, *1*.

(8) Hill, T. L. *An introduction to statistical thermodynamics*; Dover Publications: New York, 1986.

(9) Israelachvili, J. N. *Intermolecular and surface forces*, 3rd ed.; Academic press: Burlington (Mass.), 2011.

(10) Yigit, C.; Heyda, J.; Dzubiella, J. Charged patchy particle models in explicit salt: Ion distributions, electrostatic potentials, and effective interactions. *The Journal of Chemical Physics* **2015**, *143*, 064904.

(11) Lund, M. Duello - Virial Coefficient and Dissociation Constant Estimation for Rigid Macromolecules. 2025; `https://doi.org/10.5281/zenodo.15772003`.

(12) Dapkunas, J.; Timinskas, A.; Olechnovic, K.; Tomkuviene, M.; Venclovas, C. PPI3D: a web server for searching, analyzing and modeling protein–protein, protein–peptide and protein–nucleic acid interactions. *Nucleic Acids Research* **2024**, *52*, W264–W271.

(13) Perkel, J. M. Why scientists are turning to Rust. *Nature* **2020**, *588*, 185–186.

(14) Herlihy, M. P.; Wing, J. M. Linearizability: a correctness condition for concurrent objects. *ACM Transactions on Programming Languages and Systems* **1990**, *12*, 463–492.
